# Mo-W_18_O_49_/ZnIn_2_S_4_ Composites Synthesized by Metal Doping for Photocatalytic Hydrogen Evolution

**DOI:** 10.3390/molecules30071563

**Published:** 2025-03-31

**Authors:** Ruiqin Sun, Yue Liu, Jiamei Yang, Tuoya Wuren, Haochen Duan, Zhibing Tan, Shiyong Yu

**Affiliations:** 1 College of Chemistry and Chemical Engineering, Inner Mongolia University, Hohhot 010021, China; imusunruiqin@163.com (R.S.); yangjiamei333@163.com (J.Y.); wuren@imust.edu.cn (T.W.); outlook_f14ed86c8066bf58@outlook.com (H.D.); 2 China FAW Motor Corporation Limited Kinetic Energy Branch, Changchun 130011, China; 18243088624@163.com

**Keywords:** visible light, photocatalytic, Mo-W_18_O_49_, Znln_2_S_4_

## Abstract

Utilizing two or more semiconductor materials with distinct geometric and electronic energy arrangements at the nanoscale to construct heterostructures is an important means for developing high-performance catalysts for photocatalytic hydrogen evolution. In this study, ZnIn_2_S_4_ serves as the primary catalyst carrier, while Mo-W_18_O_49_ functions as the cocatalyst supported on the surface of ZnIn_2_S_4_. A series of ZnIn_2_S_4_/Mo-W_18_O_49_ heterojunction composite materials were synthesized through a straightforward hydrothermal method. The ZnIn_2_S_4_/Mo-W_18_O_49_ photocatalyst demonstrates exceptional photocatalytic hydrogen evolution activity. Notably, with a Mo-W_18_O_49_ loading of 10%, the photocatalyst achieves optimal hydrogen evolution, yielding 2592.8 μmol g^−1^, which is 31 times greater than that of pure ZnIn_2_S_4_. Further characterized results of the samples showed that loading Mo-W_18_O_49_ with an appropriate mass ratio on ZnIn_2_S_4_ can increase the electron transfer rate, which facilitates reducing the recombination probability of photo-generated electrons and holes, thus improving hydrogen evolution efficiency.

## 1. Introduction

Photocatalytic hydrogen evolution is a highly efficient and environmentally friendly method for obtaining hydrogen; therefore, numerous catalysts have been developed for improving the efficiency of this process [1,2,3,4]. To date, researchers have identified numerous photocatalysts, including metal oxides such as TiO_2_ [5,6], ZnO [7], and WO_3_ [8], as well as g-C_3_N_4_ [9,10,11,12] and metal sulfides like CdS [13], ZnS [14], and MoS_2_ [15]. Recently, ternary metal sulfide compounds such as ZnIn_2_S_4_ [16,17,18], Cd_0.9_Zn_0.1_S [19], and CaIn_2_S_4_ [20] have been extensively studied due to their unique optical band gaps and good structural stability. Among these, ZnIn_2_S_4_ is notable as the only compound with a layered structure in the metametal sulfide family. Its unit cell consists of three sub-layers: the tetrahedral ZnS_4_ surface, the octahedral InS_4_ intermediate layer, and another tetrahedral InS_4_ bottom layer. This distinctive structure offers advantages for photocatalytic applications [21].

With a hybrid metal sulfide characterized by its monolayer structure, ZnIn_2_S_4_ has garnered significant interest due to its unique architecture and photoelectric properties [22]. It has been proven to possess several advantages, including low toxicity, exceptional absorption of visible light, stability, and an appropriate band gap [23]. However, Znln_2_S_4_ exhibits a high recombination rate of photogenerated electrons and holes due to its narrow band gap, and the presence of ultra-thin Znln_2_S_4_ nanosheets may lead to spontaneous accumulation and aggregation. Consequently, the performance of Znln_2_S_4_ as a catalyst for photocatalytic hydrogen evolution needs further optimization. Although W_18_O_49_ exhibits high chemical stability [24], due to the presence of most oxygen vacancies on the surface, it demonstrates higher overpotentials, which restricts further enhancement of its catalytic performance [25]. Doping is an important method for optimizing semiconductor structures and improving their catalytic capabilities; thus, employing Mo doping to alter the electronic structure of W_18_O_49_ may effectively enhance light absorption and improve its optical properties. Consequently, the development of a viable synthesis method for Mo-doped W_18_O_49_ nanomaterials as efficient hydrogen evolution reaction (HER) catalysts warrants careful consideration. As an electron-trapping site that accelerates the surface proton reduction reaction, CoxP is grafted onto the S-type heterojunction of W_18_O_49_ZnIn_2_S_4_ (WO/ZIS-CoxP), exhibiting a strong response in the ultraviolet–visible–near-infrared region (UV–vis–NIR). This configuration provides an efficient electron transfer channel, resulting in enhanced charge separation and transfer kinetics that facilitate solar-driven hydrogen production [26,27].

In this work, Znln_2_S_4_ is utilized as the primary catalyst carrier, while Mo-W_18_O_49_ serves as the cocatalyst, which is deposited onto the surface of Znln_2_S_4_. A series of ZnIn_2_S_4_/Mo-W_18_O_49_ heterojunction composite materials were prepared using a straightforward hydrothermal synthesis method. This distinctive ZnIn_2_S_4_/Mo-W_18_O_49_ photocatalyst demonstrates excellent photocatalytic hydrogen evolution activity. Notably, with a Mo-W_18_O_49_ loading of 10%, the photocatalyst achieves optimal hydrogen evolution performance, yielding 2592.8 μmol g^−^^1^, which is 31 times greater than that of pure ZnIn_2_S_4_. Experimental characterization results indicate that the incorporation of Mo-W_18_O_49_ onto ZnIn_2_S_4_ at an appropriate mass ratio reduces charge transfer resistance, enhances the electron transfer rate, and facilitates increased electron transport, thereby improving hydrogen evolution efficiency.

## 2. Results and Discussion

### 2.1. Structural and Morphological Characterization of Photocatalysts

**Figure 1:**
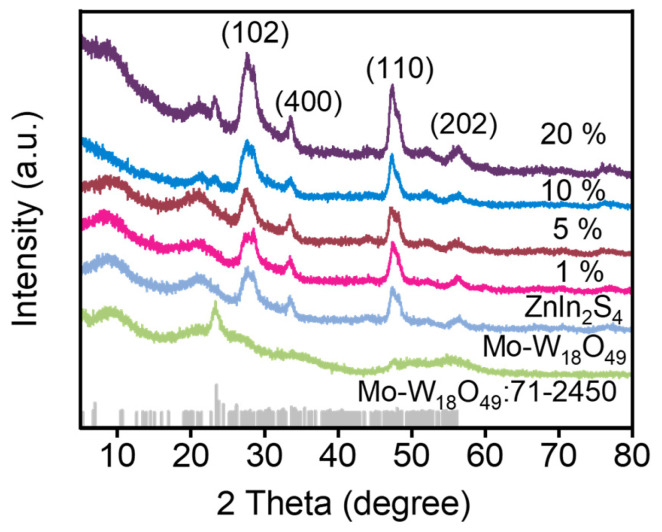
XRD patterns of Znln_2_S_4_, Mo-W_18_O_49_, and Mo-W_18_O_49_ with loading amounts of 1%, 5%, 10%, 20% on Znln_2_S_4_.

The crystallographic phases of the prepared pure phase ZnIn_2_S_4_, Mo-W_18_O_49_, and Mo-W_18_O_49_/ZnIn_2_S_4_ composites with varying compound proportions were investigated using X-ray diffraction (XRD) patterns. As illustrated in Figure 1, the pure ZnIn_2_S_4_ nanomaterials exhibit distinct diffraction peaks at 27.9°, 33.74°, 47.3°, and 55.5°, corresponding to ZnIn_2_S_4_. These peaks are attributed to the (102), (400), (110), and (202) crystallographic planes, in accordance with the standard card JCPDS No. 72-0773, and are consistent with the reported literature [28,29,30]. The spectrum of Mo-W_18_O_49_ reveals diffraction peaks at 23.4° and 26.2°, corresponding to the (010) and (-104) crystal planes, respectively. The XRD pattern of Mo-W_18_O_49_ aligns with the monoclinic crystal form, with the corresponding standard card being JCPDS No. 71-2450 [25,31]. Furthermore, when Mo-W_18_O_49_ is combined with ZnIn_2_S_4_ to form a composite material, additional diffraction peaks of Mo-W_18_O_49_ appear in the XRD spectrum alongside the original peaks of ZnIn_2_S_4_. As the loading amount of Mo-W_18_O_49_ increases, the intensity of these diffraction peaks also rises. Notably, the intensity and width of the XRD peaks for ZnIn_2_S_4_ remain unaffected by the incorporation of Mo-W_18_O_49_, indicating that the hybridization with Mo-W_18_O_49_ has minimal impact on the crystallinity and particle size of ZnIn_2_S_4_.

**Figure 2:**
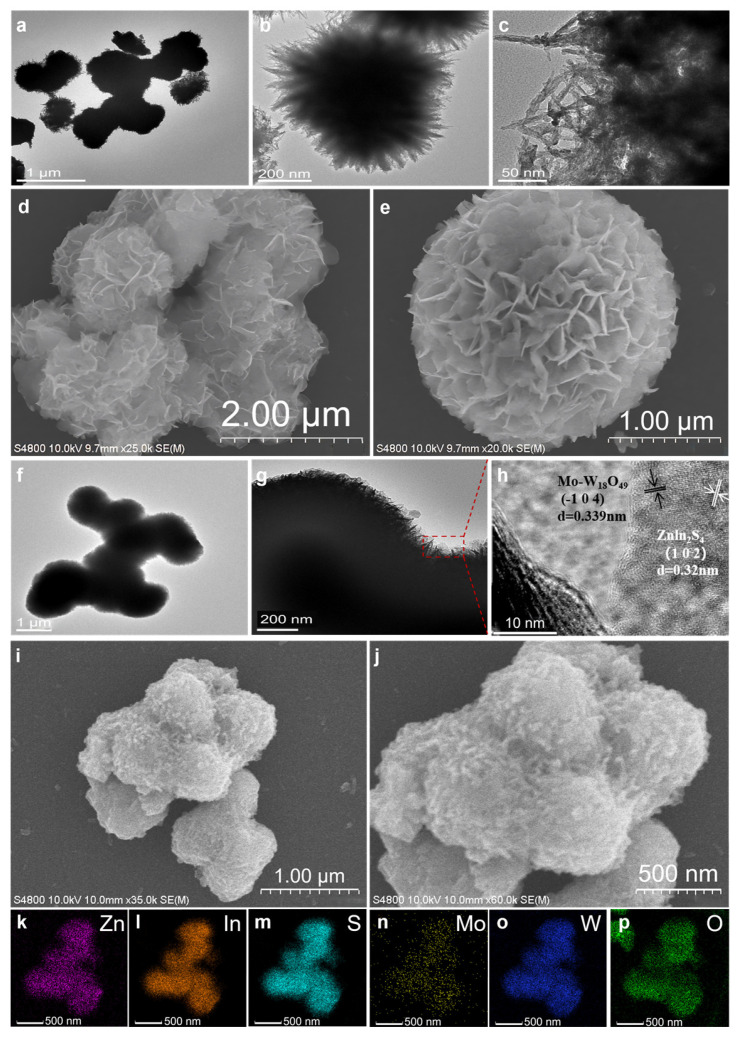
Microstructures of the proposed Mo-W_18_O_49_ and 10% Mo-W_18_O_49_/Znln_2_S_4_ composites. TEM of Mo-W_18_O_49_ (**a**–**c**), SEM of pure ZnIn_2_S_4_ (**d**,**e**), TEM of 10% Mo-W_18_O_49_/Znln_2_S_4_ (**f**,**g**), HAADF-STEM images of 10% Mo-W_18_O_49_/Znln_2_S_4_ (**h**), SEM of 10% Mo-W_18_O_49_/Znln_2_S_4_ (**i**,**j**), and EDS elemental results of Zn, ln, S, Mo, W, and O (**k**–**p**).

The morphology and microstructure of pure Mo-W_18_O_49_, 10% Mo-W_18_O_49_, and ZnIn_2_S_4_ were examined using scanning electron microscopy (SEM) and transmission electron microscopy (TEM). As illustrated in Figure 2a–c, Mo-W_18_O_49_ exhibits a sea urchin-like structure formed by the aggregation of multiple linear structures, with an average particle size of approximately 500 nm. Upon further magnification, Figure 2c reveals that Mo-W_18_O_49_ possesses a branched nanowire structure. SEM images of pure Znln_2_S_4_ in Figure 2d,e show that the Znln_2_S_4_ is a flower microsphere structure composed of two-dimensional nanosheets with a diameter of about 3 μm, and the surface of the nano-flower is relatively smooth. Figure 2f,g presents a transmission electron micrograph of 10% Mo-W_18_O_49_/ZnIn_2_S_4_; from this image, it is evident that the sea urchin-like structure of Mo-W_18_O_49_ is deposited on the surface of ZnIn_2_S_4_ nanoflowers, resulting in a smoother surface, albeit with slight agglomeration, approximately 1 micron in size.

Figure 2f displays a high-resolution transmission electron micrograph of 10% Mo-W_18_O_49_/ZnIn_2_S_4_, where two lattice fringes can be observed at 0.339 nm and 0.32 nm, corresponding to the (-104) surface of Mo-W_18_O_49_ and the (102) crystal plane of ZnIn_2_S_4_, respectively [32]. Finally, Figure 2g,h shows scanning electron microscope images of 10% Mo-W_18_O_49_/ZnIn_2_S_4_, clearly demonstrating that the sea urchin-like material is uniformly coated on the surface of the nanoflower structure. The results from the electron microscope tests indicate that Mo-W_18_O_49_ and ZnIn_2_S_4_ were successfully combined to form a composite nanomaterial via in situ deposition. An Energy Dispersive Spectroscopy (EDS) analysis was performed on the composite material to confirm the presence of its constituent elements. The EDS element map (Figure 2i–n) revealed the detection of Mo, W, O, Zn, In, and S. These elements were found to be evenly distributed across the analyzed area. Notably, the lower incorporation levels of Mo, W, and O resulted in a less uniform distribution, which aligns with the experimental observations. These findings robustly demonstrate that the one-pot solvothermal reaction can effectively synthesize 10% Mo-W_18_O_49_/ZnIn_2_S_4_ composite materials, establishing a strong intimate contact that facilitates the transfer of photogenerated carriers between ZnIn_2_S_4_ and Mo-W_18_O_49_. This intimate contact may be a critical factor contributing to the enhanced photocatalytic hydrogen evolution activity.

**Figure 3:**
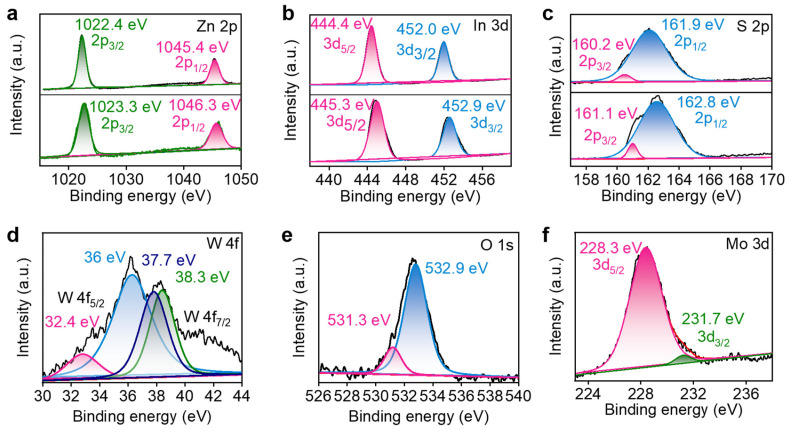
XPS spectra of 10% Znln_2_S_4_/Mo-W_18_O_49_: (**a**) Zn 2p, top: 10% Mo-W_18_O_49_/Znln_2_S_4_; bottom: Znln_2_S_4_. (**b**) In 3d, top: 10% Mo-W_18_O_49_/Znln_2_S_4_; bottom: Znln_2_S_4_. (**c**) S 2p, top: 10% Mo-W_18_O_49_/Znln_2_S_4_; bottom: Znln_2_S_4_. (**d**) W 4f. (**e**) O 1s. (**f**) Mo 3d. The curve of the actual detected electronic signal strength of the black line changing with the binding energy.

The effect of introducing Mo-W_18_O_49_ on the surface chemical state of ZnIn_2_S_4_ was investigated using X-ray photoelectron spectroscopy (XPS) characterization. Figure S1 presents the complete spectrum of 10% Mo-W_18_O_49_/ZnIn_2_S_4_. The spectrum reveals that the primary elements include Zn, In, S, Mo, W, O, and C, with no other impurities detected. Additionally, the C1s peak is observed at 284 eV. Figure 3a presents the binding energy spectrum of Zn 2p. It is evident from the figure that pure ZnIn_2_S_4_ exhibits two prominent diffraction peaks at 1023.3 eV and 1046.3 eV, corresponding to Zn 2p_3/2_ and Zn 2p_1/2_, respectively, which confirms that the valence state of Zn is +2 [23]. To determine the stoichiometry of ZnIn_2_S_4_, we performed integration of the Zn 2p, In 3d, and S 2p peaks in the XPS spectra. The atomic percentages were calculated as follows: Zn: 10.55%; In: 14.1%; S: 31.1%. Based on these values, the stoichiometry of ZnIn_2_S_4_ is approximately Zn_1.0_In_2.0_S_4.0_, which matches the expected composition. After the incorporation of Mo-W_18_O_49_, the peak positions of the two electron binding energies in the composite material shift slightly to lower binding energies, now located at 1022.4 eV and 1045.4 eV, respectively. Figure 3b illustrates the binding energy spectrum of In 3d. The pure phase ZnIn_2_S_4_ corresponds to In 3d_5/2_ and In 3d_3/2_ at binding energy positions of 445.3 eV and 452.9 eV, respectively, indicating that the In element exists as a +3 valence ion [33,34]. Similarly, the binding energy of In3d displays a blue shift following the loading of Mo-W_18_O_49_. Figure 3c presents the high-resolution XPS spectrum of the S element. The XPS spectrum of the pure phase can be fitted into two peaks, corresponding to S 2p_3/2_ and S 2p_1/2_, confirming the presence of S^2−^ [35,36]. It is apparent from the figure that the diffraction peak of the composite also exhibits a slight shift. As illustrated in Figure 3d, the high-resolution XPS spectrum of W 4f can be categorized into two pairs of peaks corresponding to W 4f_7/2_ and W 4f_5/2_, respectively. The peaks at 32.4 eV and 36 eV are associated with W 4f_5/2_, while those at 37.7 eV and 38.3 eV correspond to W 4f_7/2_. Specifically, the peak at 32.4 eV indicates the presence of +4-valent W, the emission peaks at 36 eV and 37.7 eV signify the existence of +5-valent W, and the peak at 38.3 eV represents +6-valent W [25]. In Figure 3e, the peak at 531.3 eV corresponds to the bond between tungsten and oxygen, whereas the peak at 532.9 eV is attributed to the presence of a small amount of oxygen vacancies in the material [26,27]. Figure 3f shows peaks at 228.3 eV and 231.7 eV, corresponding to the 3d_5/2_ and 3d_3/2_ states of Mo, respectively, indicating that Mo is doped in the +4 valence state [37]. The atomic percentages obtained from the peak areas are as follows: Mo: 3.53%; W: 1.14%; O: 7.75%. Based on these values, the stoichiometry of the doped oxide can be expressed as Mo_0.035_W_0.011_O_0.078_. Normalizing to the nominal composition of Mo-W_18_O_49_, this corresponds to approximately Mo_0.35_W_0.11_O_0.78_, which is close to the expected stoichiometry. Overall, the analysis confirmed the successful interaction of Mo-W_18_O_49_ and ZnIn_2_S_4_ to form S-scheme heterojunction composite photocatalyst materials. A notable shift in peak positions suggests a significant interface interaction between the two, which facilitates electron transmission. The direction of electron transfer is from ZnIn_2_S_4_ to Mo-W_18_O_49_ [38,39,40].

To further verify the formation of the composite material between the inner layer Mo-W_18_O_49_ and ZnIn_2_S_4_, Raman analysis of Mo-W_18_O_49_, ZnIn_2_S_4_, and 10% Mo-W_18_O_49_/ZnIn_2_S_4_ materials was conducted, as shown in Figure S2. The bands at 706 and 804 cm^−^^1^ correspond to the stretching modes of W-O-W, while the bands at 254 and 326 cm^−^^1^ can be attributed to the bending modes of O-W-O [25]. In the Raman spectrum of ZnIn_2_S_4_, three peaks centered at 243, 300, and 345 cm^−^^1^ were recorded, corresponding to the typical vibrational modes of ZnIn_2_S_4_: the longitudinal optical mode (LO1), the longitudinal optical mode (LO2), and the transverse optical mode (TO2) [41,42]. Additionally, in the Raman spectrum of the 10% Mo-W_18_O_49_/ZnIn_2_S_4_ material, the peak centers of ZnIn_2_S_4_ are observed to shift to 211, 197, and 250 cm^−^^1^, indicating a strong interaction between ZnIn_2_S_4_ and Mo-W_18_O_49_, which further confirms the successful formation of the composite material [26,43].

### 2.2. Photocatalytic Hydrogen Evolution Performance

**Figure 4:**
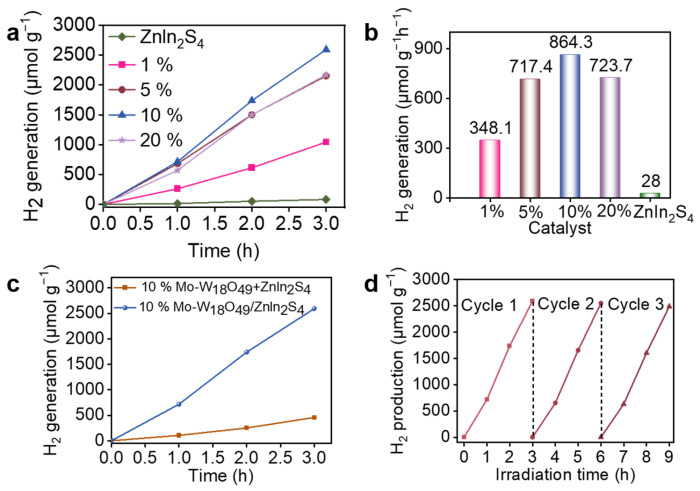
(**a**) The photocatalytic hydrogen evolution of Mo-W_18_O_49_/Znln_2_S_4_ catalysts with different loadings of Mo-W_18_O_49_ under visible light irradiation. (**b**) The photocatalytic hydrogen evolution rate of Znln_2_S_4_ and Znln_2_S_4_/Mo-W_18_O_49_ samples under light irradiation. (**c**) Comparison of photocatalytic hydrogen evolution of 10%Mo-W_18_O_49_/Znln_2_S_4_ catalyst and the same mass of Mo-W_18_O_49_ Znln_2_S_4_ mechanical mixture sample under visible light irradiation. (**d**) Cycling runs for the photocatalytic hydrogen evolution of the 10%Mo-W_18_O_49_/Znln_2_S_4_ sample under visible light irradiation.

The hydrogen evolution activity of the synthesized samples was evaluated through photocatalytic water splitting experiments. The experimental setup included a closed gas-circulation system, where the produced hydrogen was continuously circulated and sampled at regular intervals. The GC measurements were calibrated using standard hydrogen gas to ensure accuracy. The hydrogen evolution activity of the synthesized samples was evaluated through photocatalytic water splitting using lactic acid as a sacrificial agent. Figure 4a illustrates that both pure-phase ZnIn_2_S_4_ and composites loaded with varying masses of Mo-W_18_O_49_/ZnIn_2_S_4_ effectively drive the photocatalytic reduction of water to produce hydrogen under visible light irradiation, with the amount of hydrogen evolution varying among the samples. The cumulative hydrogen evolution of pure ZnIn_2_S_4_ over three hours reached only 83.5 μmol/g. However, by loading Mo-W_18_O_49_ at different mass ratios, a significant increase in hydrogen evolution was observed. At a loading of 10%, the photocatalyst exhibited optimal hydrogen evolution, yielding 2592.8 μmol g^−^^1^, which is 31 times greater than that of pure ZnIn_2_S_4_. Conversely, as the loading of Mo-W_18_O_49_ gradually increased, a decline in photocatalytic hydrogen evolution was noted, likely due to excessive Mo-W_18_O_49_ coverage on the active sites of ZnIn_2_S_4_. Figure 4b presents the photocatalytic hydrogen evolution rate of ZnIn_2_S_4_ and composite materials loaded with different mass ratios of Mo-W_18_O_49_/ZnIn_2_S_4_. The calculations indicate that the best performance achieved was 864.3 μmol g^−^^1^ h^−^^1^. These results are consistent with those shown in Figure 4a, confirming that the 10% Mo-W_18_O_49_/ZnIn_2_S_4_ composite catalyst exhibits the highest hydrogen evolution performance.

As illustrated in Figure 4c, a comparison between the 10% Mo-W_18_O_49_/ZnIn_2_S_4_ sample and the physically mixed sample of Mo-W_18_O_49_ + ZnIn_2_S_4_, both having the same mass ratio, reveals a significant difference in hydrogen evolution. The hydrogen output from the physically mixed sample is notably lower, being 5.6 times less than that of the 10% Mo-W_18_O_49_/ZnIn_2_S_4_ sample. This reduction indicates that Mo-W_18_O_49_ has deposited on ZnIn_2_S_4_ to form a heterojunction structure. Consequently, the composite photocatalyst establishes a close contact interface between Mo-W_18_O_49_ and ZnIn_2_S_4_, which facilitates charge transfer. This interaction effectively addresses the recombination issue, thereby enhancing photocatalytic hydrogen evolution activity. To evaluate the stability of the composite material, the catalyst exhibiting the best performance was selected for testing cycle performance, as illustrated in Figure 4d. The reusability of the catalyst was further assessed through three consecutive 3 h photocatalytic experiments. As shown in Figure 4d, after three cycles, hydrogen evolution remained stable, with no significant decline, achieving a total of 2488 μmol g^−1^. Compared to the first cycle, hydrogen evolution in the last two cycles exhibited a slight decrease. This reduction can be attributed to the continuous consumption of the sacrificial agent, which became insufficient during the final two cycles, leading to a decrease in hydrogen yield. Nonetheless, the photocatalyst within this composite material demonstrates commendable stability.

### 2.3. Explore the Mechanism of Photocatalytic Hydrogen Evolution

To investigate the influence of optical properties on catalyst activity, the synthesized catalysts were subjected to UV–Vis diffuse reflectance absorption spectroscopy to analyze their light absorption characteristics. As illustrated in Figure S3a, the absorption edge of ZnIn_2_S_4_ is approximately 570 nm, indicating that ZnIn_2_S_4_ is a semiconductor material with a narrow band gap and a visible light response, making it suitable for photocatalytic reactions [44]. Additionally, compared to pure ZnIn_2_S_4_, Mo-W_18_O_49_ demonstrates stronger light absorption in the visible region, consistent with literature reports [45,46]. Figure S3a further reveals that, although the visible light absorption intensity of Mo-W_18_O_49_/ZnIn_2_S_4_ composites with varying mass ratios is slightly reduced compared to pure ZnIn_2_S_4_, they exhibit the capability to absorb a broader range of visible light wavelengths. Notably, between 520 and 800 nm, the absorption intensity increases with the increasing mass ratio, resulting in a wider range of absorbed visible light wavelengths. Specifically, the composite catalyst of 10% Mo-W_18_O_49_/ZnIn_2_S_4_ demonstrates the highest visible light absorption, thereby achieving the maximum enhancement in hydrogen production performance. In Figure 4b, the bandgap values of Mo-W_18_O_49_, ZnIn_2_S_4_, and the 10% Mo-W_18_O_49_/ZnIn_2_S_4_ samples were calculated to be 1.8 eV, 1.25 eV, and 2.47 eV, respectively, based on the Tauc Plot equation. This indicates that the bandgap of the composite catalyst is significantly reduced, facilitating the absorption of light over a broader wavelength range.

**Figure 5:**
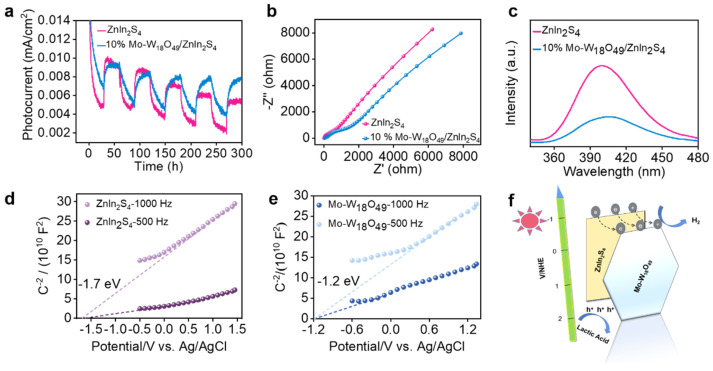
(**a**) Photocurrent density spectrum of ZnIn_2_S_4_ and 10% Mo−W_18_O_49_/ZnIn_2_S_4_. (**b**) Electrochemical impedance spectroscopy. (**c**) Photoluminescence spectrum. (**d**) Mott–Schottky plot of ZnIn_2_S_4_ and (**e**) Mo−W_18_O_49_. (**f**) Proposed photocatalytic mechanism diagram of 10% Mo-W_18_O_49_/ZnIn_2_S_4_ composites.

To investigate the enhanced efficiency of photogenerated carrier separation and transfer in 10% Mo-W_18_O_49_/ZnIn_2_S_4_ composites, we measured the performance of both pure ZnIn_2_S_4_ and the 10% Mo-W_18_O_49_ composite under visible light irradiation through multiple on/off cycles. The transient photocurrent response of the W_18_O_49_/ZnIn_2_S_4_ composite material was analyzed. As illustrated in Figure 5a, the sample generates photocurrent immediately upon illumination, which vanishes instantly when the light source is turned off. After several on/off cycles, the photocurrent response of pure ZnIn_2_S_4_ gradually decreases. In contrast, the 10% Mo-W_18_O_49_/ZnIn_2_S_4_ composite exhibits a higher and more stable transient photocurrent response, indicating that the incorporation of Mo-W_18_O_49_ enhances the photocurrent intensity. This synthesized composite material facilitates the transfer of interface electrons and holes, suggesting that photogenerated electrons are transferred more rapidly. Figure 5b presents the electrochemical impedance spectrum, where the semicircle corresponding to the 10% Mo-W_18_O_49_/ZnIn_2_S_4_ composite is the smallest and has a reduced radius compared to pure ZnIn_2_S_4_. This observation indicates a higher electron transfer rate in the 10% Mo-W_18_O_49_/ZnIn_2_S_4_ composite. Therefore, the loading of Mo-W_18_O_49_ onto ZnIn_2_S_4_ at an appropriate mass ratio reduces charge transfer resistance, increases the electron transfer rate, and enables the transport of more electrons, thereby enhancing hydrogen evolution efficiency [47,48].

Photoluminescence (PL) experiments illuminate photocatalysts with light of specific wavelengths, allowing for the acquisition of information related to electronic transitions in the emission spectrum. This further verifies the separation and transfer of electron–hole pairs. Under the excitation of incident light at a wavelength of 250 nm, the photoluminescence performance of ZnIn_2_S_4_ and 10% Mo-W_18_O_49_/ZnIn_2_S_4_ catalysts was evaluated. As shown in Figure 5c, ZnIn_2_S_4_ exhibits a pronounced peak at approximately 400 nm, which is attributed to the direct recombination of photogenerated electrons and holes resulting from intrinsic band gap excitation [49]. The figure also indicates that the 10% Mo-W_18_O_49_/ZnIn_2_S_4_ composite material displays a strong peak around 400 nm; however, the emission intensity of the composite is significantly lower than that of ZnIn_2_S_4_. This observation suggests that the strong interaction at the interface formed between the co-catalysts reduces the recombination efficiency of charges and holes, leading to fluorescence quenching.

To elucidate the photocatalytic mechanism, the Mott–Schottky technique was employed to analyze the samples and determine the sideband positions of ZnIn_2_S_4_ and Mo-W_18_O_49_, as well as their semiconductor types. As illustrated in Figure 5d,e, both ZnIn_2_S_4_ and Mo-W_18_O_49_ exhibit positive slopes in the Mott–Schottky plots, indicating that they are n-type semiconductors [50]. Additionally, the intersection with the *X*-axis allows for the determination of the sideband positions of ZnIn_2_S_4_ and Mo-W_18_O_49_. From the data presented in Figure 5d,e, it can be concluded that the flat band potentials of ZnIn_2_S_4_ and Mo-W_18_O_49_ are −1.7 eV and −1.2 eV (vs. Ag/AgCl), respectively. Previous studies have shown that the conduction band position (E_CB_) of an n-type semiconductor is determined by the flat band potential (E_fb_) [51]. Since the E_CB_ of n-type semiconductors is typically 0.1 eV more negative than E_fb_ [52], the conduction band potentials of ZnIn_2_S_4_ and Mo-W_18_O_49_ are calculated to be −1.8 eV and −1.3 eV, respectively.

Following the series of characterization results, we can further elucidate the mechanism diagram of the composite material Mo-W_18_O_49_/ZnIn_2_S_4_, as illustrated in Figure 5f. Under visible light excitation with a wavelength exceeding 420 nm, the primary catalyst ZnIn_2_S_4_ and the cocatalyst Mo-W_18_O_49_ generate photogenerated electrons and holes at their respective conduction band and valence band positions, owing to their suitable band gaps. Mott–Schottky analysis indicates that the conduction band position of ZnIn_2_S_4_ is more negative than that of Mo-W_18_O_49_. Consequently, the electrons generated in the conduction band of ZnIn_2_S_4_ are readily transferred to the conduction band of Mo-W_18_O_49_, leading to the reduction of H^+^ in H_2_O to form H_2_. Additionally, the holes generated in the valence band of Mo-W_18_O_49_ are transferred to the valence band of ZnIn_2_S_4_, where the sacrificial agent is ultimately oxidized by the holes at the valence band. This composite enhances the efficiency of photocatalytic hydrogen evolution.

## 3. Experiment

### 3.1. Materials and Methods

To synthesize Mo-W_18_O_49_, the following procedure was employed: 200 mg of WCl_6_ and 40 mg of MoCl_5_ were dispersed in 70 mL ethanol and stirred for 20 min. The resulting mixture was then transferred into a 100 mL Teflon-lined stainless steel autoclave, where it was maintained at 200 °C for 10 h. The products were subsequently washed repeatedly with deionized water and ethanol. Finally, the samples were dried at 65 °C overnight.

To synthesize ZnIn_2_S_4_/Mo-W_18_O_49_ composites, 10 mg of Mo-W_18_O_49_ powder was dissolved in 50 mL of deionized water. Appropriate amounts of Zn(NO_3_)_2_·6H_2_O and In(NO_3_)_3_·4.5H_2_O were then added to this solution, followed by the adjustment of the pH to 1 using 0.5 M HCl. A specific quantity of thioacetamide, dissolved in 20 mL of deionized water, was slowly introduced to the solution, while stirring for 1 h. The resulting mixture was transferred into 100 mL a Teflon-lined stainless steel autoclave and maintained at 160 °C for 12 h. After this period, the products were washed with deionized water and ethanol, and subsequently dried at 65 °C. The molar ratios of Mo-W_18_O_49_ to ZnIn_2_S_4_ were controlled at 1%, 5%, 10%, and 20%, labeled as 1% Mo-W_18_O_49_/Znln_2_S_4_, 5% Mo-W_18_O_49_/Znln_2_S_4_, 10% Mo-W_18_O_49_/Znln_2_S_4_, and 20% Mo-W_18_O_49_/Znln_2_S_4_, respectively.

### 3.2. Characterization

The crystal phase properties of the as-prepared products were characterized using X-ray diffraction (XRD, PANalytical B.V., Almelo, The Netherlands) with Cu-K radiation (λ = 1.5406 Å). The morphology of the samples was examined through scanning electron microscopy (SEM, HITACHI S-4800, Hitachi company, Tokyo, Japan) and transmission electron microscopy (TEM, FEI Tecnai G2 F20 STwin, FEI company, Hillsboro, OR, USA) at 200 kV. X-ray photoelectron spectroscopy (XPS) analysis was performed using a PHI 5300 ESCA instrument (Thermo Fisher Scientific (China) Co., Ltd., Shanghai, China). Additionally, electrochemical impedance spectroscopy (EIS), photocurrent measurements, and Mott–Schottky analysis were conducted on a CHI660E electrochemical workstation (Shanghai Chenhua, Shanghai, China). The photocatalyst was analyzed using a UV-Vis-NIR spectrophotometer, specifically the NEXUS-670 FT-IR spectrometer (PERKINELMER company, Waltham, MA, USA). The photocatalyst was analyzed using a Raman spectrometer, specifically the LabRAM HR Evolution model, which operates in the UV-VIS-NIR range. The excitation wavelengths were set at 514 nm, covering a spectral range from 200 to 1000 cm^−1^.

### 3.3. Photocatalytic Reaction Test

The H_2_ evolution reaction was conducted using a closed gas circulation system (Beijing Perfectlight, Perfectlight). In this system, nitrogen gas is continuously circulated, and the reactants are contained within the system throughout the reaction process. This configuration enables precise control over the reaction environment and efficient utilization of the reactants. The closed nature of the system prevents the loss of reactants or products to the external environment. A total of 50 mg of catalyst was dissolved in 90 mL of deionized water and 10 mL of lactic acid. The resulting solution was irradiated with a 300 W xenon lamp equipped with a λ > 420 nm cut-off filter. Nitrogen served as the carrier gas. Ultimately, the quantity of H_2_ produced was quantified using gas chromatography (GC7900) with a thermal conductive detector (TCD). The gas chromatography analysis was conducted using the model 9790 GC system from Zhejiang Fuli Instruments (Wenling, China).

## 4. Conclusions

Sea urchin-like Mo-W_18_O_49_ was synthesized using a two-step solvothermal method and subsequently loaded onto the ZnIn_2_S_4_ nanoflower structure to create a ZnIn_2_S_4_/Mo-W_18_O_49_ heterojunction composite photocatalyst. In this system, ZnIn_2_S_4_ serves as the primary catalyst while Mo-W_18_O_49_ acts as the co-catalyst. Under visible light irradiation, electrons generated by the primary catalyst are effectively transferred to the co-catalyst, while holes in the co-catalyst are also efficiently transferred back to the primary catalyst. This process significantly reduces the recombination rate of charge carriers. The Mo-W_18_O_49_/ZnIn_2_S_4_ heterostructure photocatalyst markedly enhances the photocatalytic performance of ZnIn_2_S_4_ for water splitting and hydrogen evolution under visible light. In the optimal 10% Mo-W_18_O_49_/ZnIn_2_S_4_ system, the hydrogen generation rate reaches 864 μmol g^−^^1^ h^−^^1^. The intimate atomic-level contact and strong interaction between ZnIn_2_S_4_ and Mo-W_18_O_49_ in the composite facilitate efficient electron transfer between the two components, thereby boosting photocatalytic activity. These findings provide a crucial foundation for the design of composite photocatalysts aimed at effective catalytic water splitting for hydrogen evolution.

## Data Availability

The original contributions presented in this study are included in the article/Supplementary Materials. Further inquiries can be directed to the corresponding authors.

## Supplementary Materials

The following supporting information can be downloaded at: https://www.mdpi.com/article/10.3390/molecules30071563/s1. Figure S1 presents the full X-ray photoelectron spectroscopy (XPS) spectrum of the 10% Mo-W_18_O_49_/ZnIn_2_S_4_ composite. Figure S2 Raman spectra of Mo-W_18_O_49_, ZnIn_2_S_4_ and 10% Mo-W_18_O_49_/ZnIn_2_S_4_. Figure S3 (a) UV-visible diffuse reflection absorption spectrogram of ZnIn_2_S_4_, Mo-W_18_O_49_ and Mo-W_18_O_49_/ZnIn_2_S_4_ with different mass ratios (b) Tauc plots of ZnIn_2_S_4_, Mo-W_18_O_49_ and 10% Mo-W_18_O_49_/ZnIn_2_S_4_.
