# Supplementary material for: Mo-W18O49/ZnIn2S4 Composites Synthesized by Metal Doping for Photocatalytic Hydrogen Evolution
Source: Molecules. 2025 Mar 31;30(7):1563. doi: 10.3390/molecules30071563 (PMC11990356; doi:10.3390/molecules30071563)
Supplement: Supplementary file 1 [file molecules-30-01563-s001.zip › molecules-3517226-supplementary.pdf]

Mo-$W_{18}O_{49}$/$ZnIn_2S_4$ composites synthesized by metal doping for photocatalytic hydrogen evolution

Ruiqin Sun[1,†], Yue Liu[2,†], Jiamei Yang[1], Tuoya WuRen[1], Haochen Duan[1], Zhibing Tan[1,*] and Shiyong Yu[1,*]

[1] College of Chemistry and Chemical Engineering, Inner Mongolia University, Hohhot, 010021, China
[2] China FAW Motor Corporation Limited Kinetic Energy Branch, Changchun, 130011, China
* Correspondence: zhibingtan@imu.edu.cn (Z.T.); syyunano@imu.edu.cn (S.Y.)
† These authors contributed equally to this work.

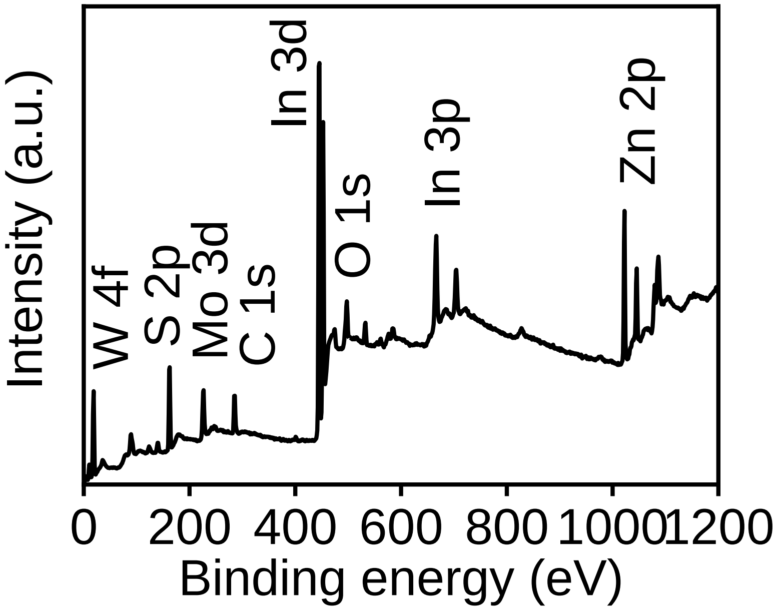

Figure S1 presents the full X-ray photoelectron spectroscopy (XPS) spectrum of the 10% Mo-$W_{18}O_{49}$/$ZnIn_2S_4$ composite.

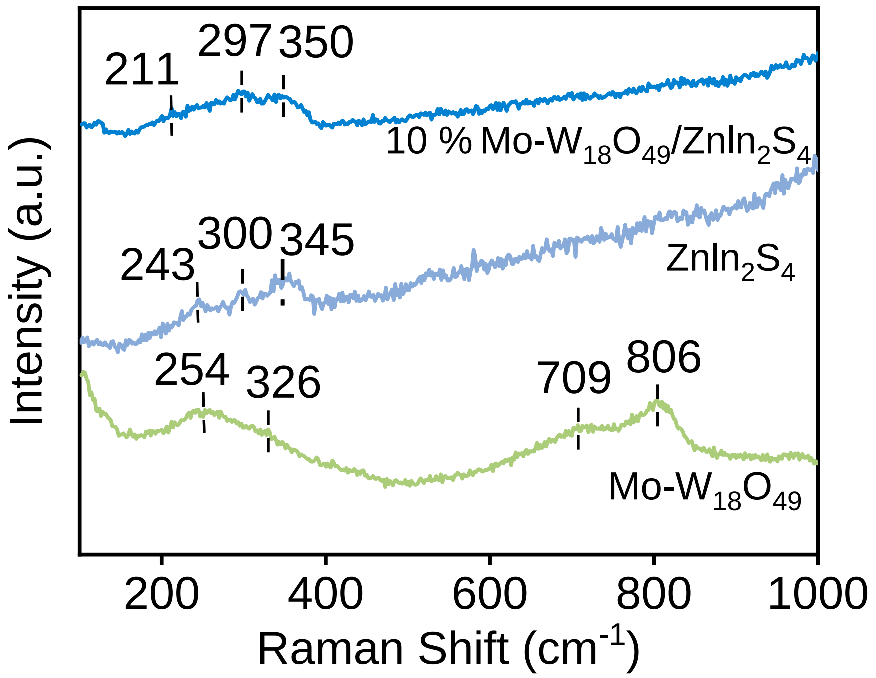

Figure S2 Raman spectra of Mo-$W_{18}O_{49}$, $Znln_2S_4$ and 10% Mo-$W_{18}O_{49}$/$ZnIn_2S_4$.

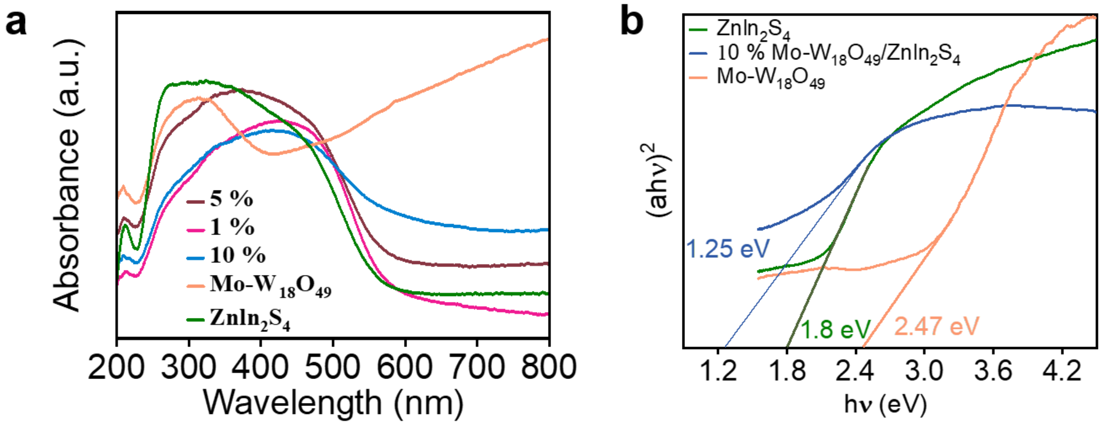

Figure S3 (a) UV-visible diffuse reflection absorption spectrogram of $ZnIn_2S_4$, Mo-$W_{18}O_{49}$ and Mo-$W_{18}O_{49}$/$ZnIn_2S_4$ with different mass ratios (b) Tauc plots of Znln2S4, Mo-$W_{18}O_{49}$ and10%Mo-$W_{18}O_{49}$/$ZnIn_2S_4$.
